# Measurement of the ^85^Kr specific activity in the GERDA liquid argon

**DOI:** 10.1140/epjc/s10052-025-14135-8

**Published:** 2025-05-12

**Authors:** M. Agostini, A. Alexander, G. Araujo, A. M. Bakalyarov, M. Balata, I. Barabanov, L. Baudis, C. Bauer, S. Belogurov, A. Bettini, L. Bezrukov, V. Biancacci, E. Bossio, V. Bothe, R. Brugnera, A. Caldwell, S. Calgaro, C. Cattadori, A. Chernogorov, P.-J. Chiu, T. Comellato, V. D’Andrea, E. V. Demidova, N. Di Marco, E. Doroshkevich, M. Fomina, A. Gangapshev, A. Garfagnini, C. Gooch, P. Grabmayr, V. Gurentsov, K. Gusev, J. Hakenmüller, S. Hemmer, W. Hofmann, J. Huang, M. Hult, L. V. Inzhechik, J. Janicskó Csáthy, J. Jochum, M. Junker, V. Kazalov, Y. Kermaïdic, H. Khushbakht, T. Kihm, K. Kilgus, I. V. Kirpichnikov, A. Klimenko, K. T. Knöpfle, O. Kochetov, V. N. Kornoukhov, P. Krause, V. V. Kuzminov, M. Laubenstein, M. Lindner, I. Lippi, A. Lubashevskiy, B. Lubsandorzhiev, G. Lutter, C. Macolino, B. Majorovits, W. Maneschg, G. Marshall, M. Misiaszek, M. Morella, Y. Müller, I. Nemchenok, M. Neuberger, L. Pandola, K. Pelczar, L. Pertoldi, P. Piseri, A. Pullia, C. Ransom, L. Rauscher, M. Redchuk, S. Riboldi, N. Rumyantseva, C. Sada, S. Sailer, F. Salamida, S. Schönert, J. Schreiner, A.-K. Schütz, O. Schulz, M. Schwarz, B. Schwingenheuer, O. Selivanenko, E. Shevchik, M. Shirchenko, L. Shtembari, H. Simgen, A. Smolnikov, D. Stukov, S. Sullivan, A. A. Vasenko, A. Veresnikova, C. Vignoli, K. von Sturm, T. Wester, C. Wiesinger, M. Wojcik, E. Yanovich, B. Zatschler, I. Zhitnikov, S. V. Zhukov, D. Zinatulina, A. Zschocke, K. Zuber, G. Zuze, GERDA Collaboration*

**Affiliations:** 1 INFN Laboratori Nazionali del Gran Sasso, Assergi, Italy; 2 INFN Laboratori Nazionali del Gran Sasso and Gran Sasso Science Institute, Assergi, Italy; 3 INFN Laboratori Nazionali del Gran Sasso and Università degli Studi dell’Aquila, L’Aquila, Italy; 4 INFN Laboratori Nazionali del Sud, Catania, Italy; 5 Institute of Physics, Jagiellonian University, Kraków, Poland; 6 Institut für Kern- und Teilchenphysik, Technische Universität Dresden, Dresden, Germany; 7 Joint Institute for Nuclear Research, Dubna, Russia; 8 European Commission, JRC-Geel, Geel, Belgium; 9 Max-Planck-Institut für Kernphysik, Heidelberg, Germany; 10 Department of Physics and Astronomy, University College London, London, UK; 11 INFN Milano Bicocca, Milan, Italy; 12 Dipartimento di Fisica, Università degli Studi di Milano and INFN Milano, Milan, Italy; 13 Institute for Nuclear Research of the Russian Academy of Sciences, Moscow, Russia; 14 Institute for Theoretical and Experimental Physics, NRC “Kurchatov Institute”, Moscow, Russia; 15 National Research Centre “Kurchatov Institute”, Moscow, Russia; 16 Max-Planck-Institut für Physik, Munich, Germany; 17 Physik Department, Technische Universität München, Munich, Germany; 18 Dipartimento di Fisica e Astronomia, Università degli Studi di Padova, Padua, Italy; 19 INFN Padova, Padua, Italy; 20 Physikalisches Institut, Eberhard Karls Universität Tübingen, Tübingen, Germany; 21 Physik-Institut, Universität Zürich, Zurich, Switzerland; 22 Present Address: Duke University, Durham, NC USA; 23 Present Address: Semilab Zrt, Budapest, Hungary; 24 Present Address: Nuclear Science Division, Berkeley, USA; 25 Present Address: IRFU, CEA, Université Paris-Saclay, Gif-sur-Yvette, France; 26 NRNU MEPhI, Moscow, Russia; 27 Moscow Institute of Physics and Technology, Moscow, Russia; 28 Dubna State University, Dubna, Russia; 29 Max-Planck-Institut für Kernphysik, Heidelberg, Germany

## Abstract

The radioactive isotope ^85^Kr is found in significant quantities in the atmosphere largely due to nuclear industry. Its ${\upbeta }$-decay with a half-life of 10.7 years and a Q-value of 687 keV is a dangerous background source for low-threshold noble gas and liquid detectors, which distill their detector medium from air. The Gerda experiment was operating high-purity germanium detectors immersed in a clean liquid argon bath deep underground to search for neutrinoless double beta decay with unprecedented sensitivity. The ^85^Kr specific activity in the liquid argon at the start of the second phase of the experiment has been determined to be $(0.36 \pm 0.03)$ mBq/kg through an analysis of the full subsequent data set that exploits the excellent ${\upgamma }$-ray spectroscopic capabilities of Gerda.

## Introduction

The ${\upbeta }$-decay of ^85^Kr ($T_{1/2} = 10.7$ yr, $Q_{{\upbeta }}= 687$ keV) is a background in low-energy-threshold detectors employing noble gases or liquids cryogenically distilled from the atmosphere as detector medium [1].

The presence of ^85^Kr in the atmosphere is largely anthropogenic: being a nuclear fission product, it can reach the atmosphere in spent nuclear fuel reprocessing plants, nuclear weapon tests or accidents. As a result, the average atmospheric ^85^Kr specific activity^1^ has steadily increased since the beginning of the nuclear industry era to an average global value of 1–2 Bq/m^3^ [2–4]. Moreover, concentrations are typically higher nearby nuclear reprocessing facilities and generally higher in the northern hemisphere than in the southern hemisphere. Specific meteorological conditions also induce regional differences.

Since the ^85^Kr activity in distilled gases or liquids correlates to that in air at the production facility and at distillation time, its initial value can significantly vary across different batches. Moreover, ^85^Kr can leak from the atmosphere into the experiment over time, depending on the detector technology. The WARP collaboration has reported an activity of $(0.12 \pm 0.09)$ Bq/kg in atmospheric liquid argon (LAr) by directly constraining the ^85^Kr ${\upbeta }$-decay spectrum [5]. Using the same method, the DarkSide collaboration has measured an unexpectedly high activity of $(2.05 \pm 0.13)$ mBq/kg in underground sourced LAr, potentially due to atmospheric leaks or from natural fission underground [6]. Liquid xenon (LXe) experiments typically remove ^85^Kr by cryogenic distillation [7, 8] or gas chromatography [9, 10]. A concentration of natural krypton (^nat^Kr) of 480 ppq (mol/mol) in LXe (corresponding to 0.14 $\upmu$Bq/kg of ^85^Kr, assuming a ^85^Kr/^nat^Kr abundance of $2 \cdot 10^{-11}$) has been measured using rare gas mass spectroscopy (RMGS) [11] in the XENONnT detector after filling and reduced to $(56 \pm 36)$ ppq through a krypton distillation column [8]. 144 ppq (g/g) of ^nat^Kr (27 nBq/kg of ^85^Kr) have been measured through a liquid nitrogen cold trap in the LZ detector after purification through gas chromatography [12].

**Fig. 1:**
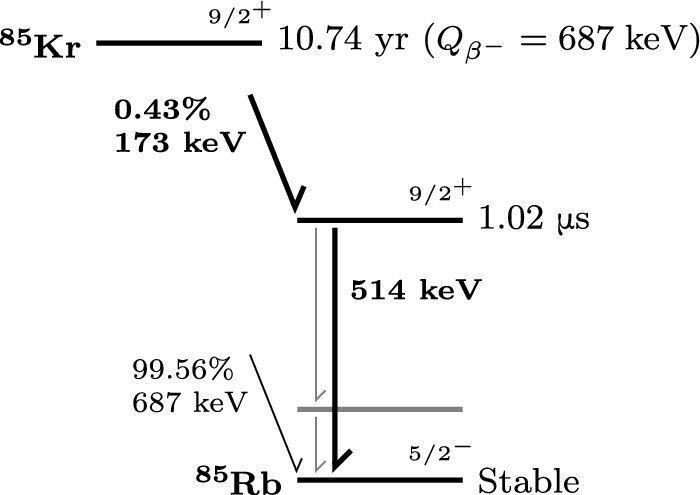
Simplified ^85^Kr decay scheme [13]. The decay channel studied in this work is highlighted in bold

^85^Kr decays to ^85^Rb via ${\upbeta }$-decay with a half-life of 10.7 yr. In 0.43% of the cases an excited ^85^Rb nucleus is produced, which de-excites to the ground state by emitting a 514 keV ${\upgamma }$-ray with a half-life of 1 $\upmu$s [13]. A simplified decay scheme is shown in Fig. 1.

The GERmanium Detector Array (Gerda) experiment operated high-purity germanium (HPGe) detectors in a cryogenic bath of atmospheric LAr. Full absorption of 514 keV ${\upgamma }$-rays in germanium following ^85^Rb de-excitations produces a narrow peak-like signature in the Gerda HPGe detectors, thanks to their excellent energy resolution [14] and the fact that ${\upbeta }$ particles are typically fully absorbed in argon. To measure the activity of ^85^Kr nuclei in LAr, the number of events in the full energy peak (FEP) can be therefore extracted with a simple analytical model and then corrected by the detection efficiency, determined through a Monte Carlo simulation.

This article is organized as follows: we start by giving a summary of the experimental setup in Sect. 2, then proceed to a description of the data set selection in Sect. 3. The determination of the ^85^Kr decay signature and conversion factor between observed counts and ^85^Kr activity at filling time through Monte Carlo simulations is presented in Sect. 4. The statistical techniques employed to analyze the data, including likelihood function and fit model, are detailed in Sect. 5, followed by an assessment of systematic uncertainties in Sect. 6. The final results on the ^85^Kr activity and their interpretation are discussed in Sects.7 and 8, respectively.

## The GERDA experiment

The Gerda experiment, decommissioned at the beginning of 2020, was primarily aimed at searching for the lepton number violating neutrinoless double beta $0{\upnu } {\upbeta }{\upbeta }$decay [15] deep underground at the Laboratori Nazionali del Gran Sasso (LNGS) of the Istituto Nazionale di Fisica Nucleare (INFN), in Italy. HPGe detectors, isotopically-enriched in ^76^Ge at $\sim$88%, were arranged in a closely-packed, low-background string array and operated in a 64 m^3^ LAr cryostat [16], inside a 590 m^3^ water tank instrumented with photomultiplier tubes (PMTs). The latter, together with scintillating panels placed at the top of the experiment, constituted a passive and active shield against laboratory and cosmic backgrounds [17]. The cryostat was filled between November and December 2009 with 5.0-grade (i.e. 99.999% pure) LAr, distilled from the atmosphere at the Linde 2 facility in Trieste (Italy). At LNGS the LAr was transferred from the Linde tanker via a 6.3 m^3^ storage tank and an about 30 m long vacuum-insulated pipe with an ultra-fine filter at the end in the cryostat (see section 4.1 of Ref. [16] for more details); no further distillation or purification was done. After this first major filling, only few minor top-ups took place during the lifetime of the experiment. The fraction of the argon gas volume was about 5% of the LAr volume. In consideration of the measures taken to seal the LAr from the atmosphere, in order to preserve its optical properties in the absence of an online purification system, a ^85^Kr re-contamination during data taking is not expected. Moreover, the in-situ ^85^Kr production rate due to cosmic rays or spontaneous fission of ^238^U is negligible at LNGS.

In the second phase of the experiment (Phase II), the array consisted of 10 semi-coaxal (Coax) detectors (including 3 detectors with natural isotopic abundance) and 30 Broad Energy Germanium (BEGe) detectors, arranged in a seven-string layout. The detector array was surrounded by a scintillation light readout instrumentation made by two sub-systems: low-activity PMTs and wavelength-shifting (WLS) fibers coupled to silicon photomultipliers [18, 19]. Each HPGe detector string was enclosed in a nylon cylinder to limit the collection of radioactive potassium ions on the detector surface [20]. A cylindrical copper shroud was shielding the array against ^222^Rn emanating from e.g. the cryostat walls. A detailed description of the Phase II experimental setup has been published in [18]. The three natural HPGe detectors and one Coax detector were removed and five new inverted-coaxial (IC) detectors were deployed during a hardware upgrade in spring 2018 [21]. The coverage and radio-purity of the WLS fibers was also improved. The energy calibration of the HPGe detectors was performed during dedicated weekly calibration runs in which the detectors were exposed to three ^228^Th sources [14].

**Fig. 2:**
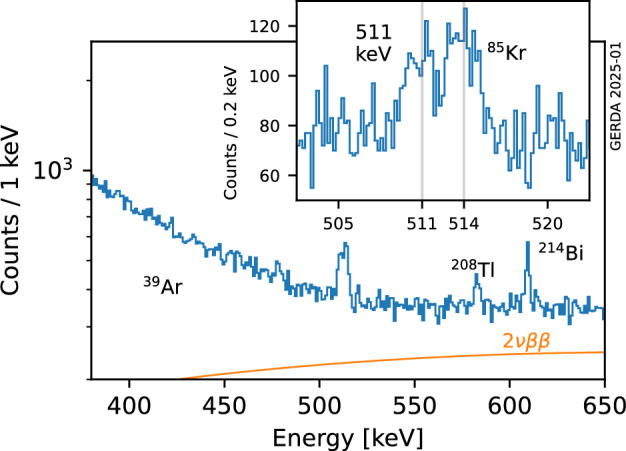
The energy spectrum of the Gerda data around the region of interest at the ^85^Kr FEP (514 keV), with labels indicating the prominent spectral features and the expected contribution from the 2${\upnu } {\upbeta }{\upbeta }$ decay in orange. The inset shows a zoom around the region of interest with a finer binning (0.2 keV), in linear *y*-scale

**Fig. 3:**
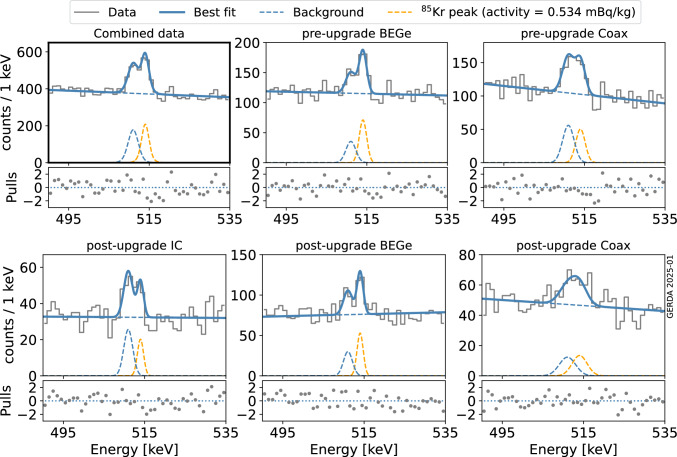
The best-fit model superimposed to data. The highlighted top-left panel shows a combination of the entire Gerda Phase II data set. The remaining panels separately show data from different detector types (BEGe, Coax and IC) and before or after the May 2018 hardware upgrade. The continuum, the 511 keV peak and the ^85^Kr FEP are plotted separately with dashed lines. The difference between data and best fit model for each bin, normalized by standard deviation expected from Poisson statistics, is shown in a panel below each spectrum

## Data selection

To constrain the ^85^Kr activity, we consider the full Phase II physics data set, corresponding to data taken from December 2015 to November 2019 with a short interruption in May 2018 due to the hardware upgrade. The accumulated HPGe exposure usable for analysis is 105.5 kg yr (61.4 kg yr before and 44.1 kg yr after the upgrade). Data from detectors made from natural germanium is discarded due to detector operational instabilities and low exposure contribution.

The standard HPGe detector digital signal processing pipeline [22], including a zero-area-cusp filter for energy reconstruction [23], are applied. Non-physical and pile-up events are identified and removed with an estimated acceptance rate of (physical) events of more than 99.9% [15]. Events classified as muon-like, as well as those characterized by energy depositions in multiple detectors are removed from the data set. Data from the LAr instrumentation and HPGe signal pulse shapes have not been included in this analysis, as they do not improve the overall accuracy in determining the ^85^Kr activity. This is due to the large uncertainties in the signal acceptance efficiencies of the LAr veto and HPGe pulse shape discrimination (PSD) at these energies.

Due to different detector properties, e.g. energy resolution and efficiency, and changes in the detector configuration during the upgrade, the data is divided into five data sets: pre-upgrade BEGe, pre-upgrade Coax, post-upgrade BEGe, post-upgrade Coax and post-upgrade IC.

The energy spectrum of the combined data set is shown in Fig. 2. The main backgrounds contributing in the $\sim$0.5 MeV energy region and below are the decay of ^39^Ar ($Q_{{\upbeta }}= 565$ keV) in LAr, the $2{\upnu } {\upbeta }{\upbeta }$ decay of ^76^Ge, ^42^Ar decays in LAr and decays of ^40^K, ^238^U and ^232^Th chain isotopes in structural materials. A detailed model of the Gerda Phase II energy spectrum has been published in [24]. The inset shows a zoom in the region around 514 keV with a finer binning. A prominent peak-like structure is visible, constituted by ^85^Rb de-excitation 514 keV ${\upgamma }$-rays fully absorbed in the active volume of the HPGe detectors and 511 keV ${\upgamma }$-rays from positron annihilation and the ^208^Tl decay cascade. The positrons originate from pair production of high energy gamma rays. The excellent energy resolution of the Gerda detectors [14], in particular BEGe and IC post-upgrade, results into a separation between the two event populations (Fig. 3).

Data from Gerda Phase I is not considered in this analysis, as the energy resolution of HPGe detectors was not at the level required to disentangle the 511 keV and the 514 keV peaks [25].

## ^85^Kr signature and conversion factor

**Fig. 4:**
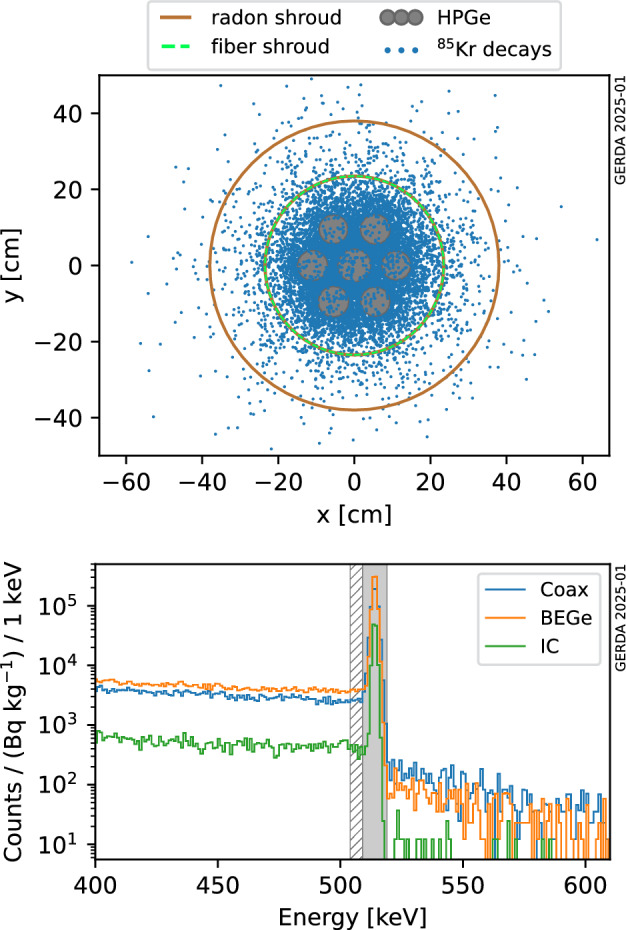
Top panel: horizontal spatial distribution of a sample of simulated ^85^Kr decays associated with an energy deposition of 514 keV in the HPGe array. Key structural components (the radon shroud, the WLS fiber shroud and the detectors) are pictorially shown. Bottom panel: expected signature of the decay in the Gerda energy spectrum, for each detector type, normalized by ^85^Kr specific activity at the start of Gerda Phase II. 10^10^ total decays have been simulated in a LAr cylinder (see text). The energy windows used to calculate the full-energy peak efficiency are shown in gray (see text)

The Geant4-based [26–28] application MaGe [29], which implements the full Gerda Phase II experimental setup to a high level of detail, is used to simulate ^85^Kr decays and track their products. The decay vertices are uniformly distributed in the LAr volume enclosed by a cylinder (radius 70 cm, height 180 cm, $m_\text {LAr} = 3835$ kg) centered at the HPGe array. The dimensions of such cylinder are significantly larger than the absorption length of 514 keV ${\upgamma }$-rays in LAr (of about 10 cm) and do not bias the energy distribution of the HPGe detector hits. Events that deposit energy in the HPGe detectors are stored on disk for further offline processing. The top panel of Fig. 4 shows the *xy* spatial distribution of a sample of simulated ^85^Kr decays in which the 514 keV ${\upgamma }$-ray is fully absorbed in the active volume of the detectors. A representation of the salient features of the setup (HPGe detectors, WLS fiber curtain and radon shroud) is overlaid.

**Fig. 5:**
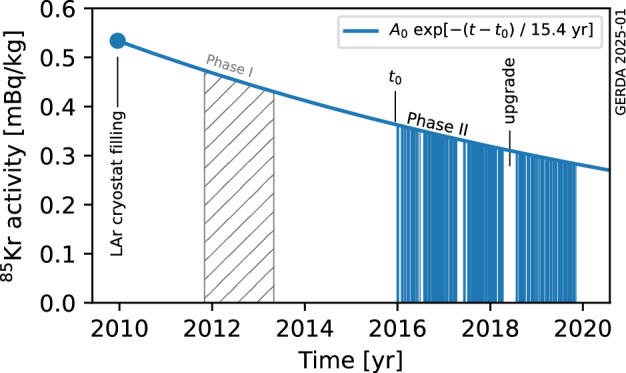
Visualization of the exponential decrease of the ^85^Kr activity (continuous blue line) during the Gerda data taking. Gerda Phase II data taking (blue areas) started at $t_0 = \text {25 December 2015}$. The time of LAr cryostat filling, 17 December 2009, and the Phase I data taking period (dashed area), not considered in this work, are indicated. The activity $A_0$ is obtained by a fit to the Gerda Phase II data (see Sect. 7)

At the Monte Carlo event post-processing stage, the operational status of each individual detector in the considered data taking period is taken into account. Since the ^85^Kr half-life is comparable to the Gerda lifetime, the exponential decrease of its activity:

$$\begin{aligned} A(t) = A_0 \exp [- \lambda (t - t_0)] , \end{aligned}$$

where $A_0 = A(t_0)$ is the ^85^Kr activity at time $t_0$ and $\lambda = \ln (2) / 10.7$ year, is also taken into account. The procedure is illustrated in Fig. 5. The blue solid line represents the ^85^Kr decay curve while each blue-filled region corresponds to a physics data taking run. The largest silent period corresponds to the hardware upgrade works.

In addition to the hardware settings, a model of the HPGe detector active volume is applied, as detailed in [24]. The thickness of the dead layer at the n$^+$ electrode, measured during detector characterization before deployment [30], is varied at the post-processing stage according to its uncertainty to estimate the impact on the conversion factor (see Sect. 6).

After post-processing, probability density functions (pdfs) of the energy deposited in HPGe detectors are obtained from the simulated event sample for each analysis data set. The bottom panel of Fig. 4 shows for the three Gerda detector types the expected counts in Phase II for 1 Bq/kg ^85^Kr in 1 keV binning. The spectra are smeared with effective energy resolution curves corresponding to the Phase II data set [14]. By scaling these pdfs by the ^85^Kr activity (in Bq/kg) $A_0$, the expected event distribution detected by Gerda is obtained. It is characterized by a prominent peak at 514 keV, its Compton continuum, and significantly fewer high-energy events resulting from the simultaneous, unlikely detection of a ${\upbeta }$-particle that reaches the HPGe active volume and a delayed ^85^Rb de-excitation ${\upgamma }$-ray. The half-life of the excited nucleus (1 $\upmu$s) is compatible with the time scale of HPGe signals, leading to the observation of a single pulse carrying the summed energy from the ${\upbeta }$-particle and the ${\upgamma }$-ray.

Table 1 reports the conversion factor $\xi$ of 514 keV FEP events which is defined as: the number of observed events N for each analysis data set is $N = A_0\,\xi$. It is computed from the pdfs as the difference between the integral in the signal window [509, 519] keV (solid gray region in Fig. 4) and the background window [504, 509] keV (hatched gray region), taking into account the respective widths. Note that, as the signal-to-background ratio is very high, the actual window choice does not affect the results. The uncertainty includes the mentioned HPGe detector active volume effects, and has been estimated by varying the dead-layer thicknesses in their experimental uncertainty.

**Table 1:** Summary of the effective energy resolution (full width at half maximum) at 514 keV and conversion factor $\xi$ ($\xi > 1$, see text for exact definition) for each analysis data set, with start of the Gerda Phase II data taking $t_0 = \text {25 December 2015}$. Reported uncertainties on $\xi$ include active volume effects

Dataset	FWHM	$\xi /10^5$
	(keV)	(Bq/kg)$^{-1}$
*Before upgrade*		
BEGe	2.2 ± 0.2	$4.88 \pm 0.14$
Coax	2.7 ± 0.2	$4.44 \pm 0.34$
*After upgrade*		
BEGe	1.8 ± 0.1	$1.89 \pm 0.05$
Coax	3.3 ± 1.3	$2.98 \pm 0.09$
IC	2.2 ± 0.1	$1.137 \pm 0.008$

## Analysis method

The ^85^Kr activity is extracted through maximum likelihood estimation from the combined analysis of the five data sets, in the energy window [490, 535] keV using a 1 keV binning. Given the Gerda energy resolution, this window fully contains the ^85^Kr FEP at 514 keV, the signal of interest. It has been checked that the choice of bin width does not have any influence on the results by repeating the analysis with smaller bins.

The sum of the backgrounds responsible for the continuum event distribution (^39^Ar, $2{\upnu } {\upbeta }{\upbeta }$ and Compton scatters) discussed in Sect. 3 is modeled as a linear function, which provides a good approximation in the analysis window. The signal is modeled with a Gaussian peak, centered at the expected energy of 514 keV and with a width given by the data set energy resolution (reported in Table 1). The latter is determined by combining all ^228^Th calibration data, as detailed in [14]. A second Gaussian peak is introduced to describe the sum of the e$^+$e$^-$ annihilation peak and the ^208^Tl ${\upgamma }$-peak. It is centered at the expected energy of 511 keV, but an additional broadening factor *f* is introduced to account for possible deviation in the peak width from the reference energy resolution due to Doppler effects in e$^+$e$^-$ annihilation. This width $\sigma$ can therefore be written as:

1 $$\begin{aligned} \sigma ^2(E_\text {ann}, f) = {(\text {FWHM}(E_\text {ann})/2.355)}^2 + f^2 \,, \end{aligned}$$

where $E_\text {ann}=511$ keV, $2.355 \approx 2\sqrt{2\ln 2}$ and the (FWHM) is calculated from the effective energy resolution curves [14].

The full likelihood reads as follows:

2 $$\begin{aligned} \mathcal {L}(\text {data} \,|\, A_0, \vec {\vartheta } ) = \prod _i^\text {ds} \prod _j^\text {bins} \operatorname {Pois}(\nu _{ij} \,|\, \mu _{ij}(A_0, \vec {\vartheta }_i)) \times \operatorname {Pull}(\vec {\vartheta }_i) \,, \end{aligned}$$

where $\text {Pois}(\nu \,|\, \mu )$ is the Poisson distribution pdf, the products run over the data sets *i* and bins *j* and $\operatorname {Pull}$ denotes additional pull terms described in the following. The likelihood depends on the ^85^Kr activity $A_0$, which is a common parameter among all the 5 data sets and the only parameter of interest, and on a set of nuisance parameters $\vec {\vartheta }$ that are data set specific and affect both the signal and background distributions. Finally, $\nu _{ij}$ denotes the number of observed events in the data set *i* and bin *j*, and $\mu _{ij}$ is the expectation value for the same data set and bin. The latter is given by the sum of signal and background contributions in that bin, $bin, \mu _ij = b_{ij} + s_{ij}$. The expected number of signal events can be written as:

$$\begin{aligned} s_{ij} = A_0 \, \xi _i \, \int _{\Delta E_j} dE \, \mathcal {N}(E \,|\, E_K+ \delta _i(E_K), \sigma ^2_i(E_K)) , \end{aligned}$$

where $\mathcal {N}(E \,|\, \mu , \sigma ^2)$ is the normal distribution and $E_K= 514$ keV. The expression depends on the ^85^Kr activity $A_0$, the ^85^Kr FEP conversion factor $\xi _i$ (defined in Sect. 4), the energy scale systematic bias term $\delta _i(E_K)$, and the energy resolution

$$\begin{aligned} \sigma _i(E_K) = \text {FWHM}{(E_K)}_i \,/\, 2.355 . \end{aligned}$$

The expected number of background events can be written as:

$$\begin{aligned} b_{ij}&= \int _{\Delta E_j} dE \, \big [ N^b_i \, \operatorname {Pol1}(E, \vec {p}_i) \\&\quad + N^s_i \, \mathcal {N}(E \,|\, E_\text {ann}+ \delta _i(E_\text {ann}), \sigma ^2_i(E_\text {ann}, f)) \big ] \;, \end{aligned}$$

i.e. the sum of a linear contribution $N^b_i \, \text {Pol1}(E, \vec {p}_i)$, which depends on the normalization of the linear distribution $N^b_i$ and its parameters $\vec {p}_i$, both data set specific, and a normal contribution, describing the 511 keV peak, which depends on the normalization $N^s_i$, the energy scale bias $\delta _i(E_\text {ann})$, and the broadened energy resolution given by Eq. (1). All the parameters entering this last normal term are data set specific, except for the broadening factor *f*, which is kept the same for all the data sets. We verified that the first-order polynomial function describes the continuum in this energy region well and that a second-order polynomial function does not improve the fit.

A product of normal pull terms

$$\begin{aligned} \operatorname {Pull}(\vec {\vartheta }_i) = \prod _k \mathcal {N}(\theta _{ik} \,|\, \ldots ) \end{aligned}$$

is included in the likelihood in Eq. (2) to constrain some of the nuisance parameters, namely the conversion factor $\xi _i$, the energy scale bias $\delta _i$ and the energy resolution $\sigma _i$, according to their expected distribution. These will be discussed in more detail in Sect. 6. All the other nuisance parameters, namely the parameters of the linear background, the number of events in the 511 keV peak, and the broadening, are free and are left unconstrained. To estimate the uncertainty on the parameters of interest, the profile likelihood is used.

## Systematic uncertainties

In this section, the uncertainties affecting the conversion factor $\xi$ are first discussed and evaluated. They can be categorized into: data quality cuts, cryostat top-ups, and HPGe active volume. Uncertainties affecting the energy scale and resolution are discussed in the last part of the section.

*Data quality cuts* Physical events with an energy in the region of interest are accepted with an efficiency larger than 99.9% [15]. If both the ${\upbeta }$-particle and the subsequent 514 keV ${\upgamma }$-ray from ^85^Kr decay reach the active volume of the same HPGe detector, the delay of the two pulses (the half-life of the excited ^85^Rb state is $\sim$1 $\upmu$s) in the digitized HPGe signal could be, in principle, large enough to trigger the pile-up rejection algorithm, which is not modeled in Monte Carlo simulations. In practice, the rate of such coincident detection in the ^85^Kr FEP region is so low (due to the much lower detection efficiency of ^85^Kr decay ${\upbeta }$-particles, see Fig. 4) that the impact on the detection efficiency is negligible.

*Cryostat top up* The Gerda cryostat has been periodically refilled with small amounts of LAr between 2009 and 2018. This argon might have had a different ^85^Kr activity, compared to that in the cryostat, and might have therefore had an impact on the existing contamination. We estimated a mass of additional argon of roughly 2.5 tons, corresponding to less than 3% of the total argon volume, additionally deployed in the experiment. The impact on the estimated ^85^Kr activity has been evaluated by assuming that the whole amount was deployed during the hardware upgrade works (see Fig. 5), a conservative assumption that largely corresponds to reality. In such a scenario, the initial ^85^Kr activity would be overestimated by less than 2%, within this analysis.

*HPGe active volume* Uncertainties on the size of the HPGe active volume affect the conversion factor $\xi$. Typical sizes of the detector dead layers are 1–2 mm known with an uncertainty of 5–30% [30]. The contribution to $\xi$ varies according to the data set: the active volume of Coax detectors is poorly known, and BEGe detectors suffer from a large uncertainty too, due to dead-layer growing effects. The IC active volume is, on the other hand, better constrained. To determine the impact on each of the $\xi _i$, Monte Carlo simulations have been re-processed while varying the dead-layer model within the respective uncertainties. The uncertainties reported in Fig. 1 include these effects, which contribute with about 3% in case of BEGe detectors, 8% in case of Coax and less than 1% in case of IC.

*Energy scale and resolution* The uncertainty on energy calibration and resolution, parametrized in Eq. (2) by pull terms on the $\delta _i$ and $\sigma _i$ nuisance parameters, respectively, can be estimated based on $^{228}$Th calibration data. Such an evaluation has been carried out by focusing at 2 MeV, in the context of the $0{\upnu } {\upbeta }{\upbeta }$ decay analysis [14]. In this work, we base our estimate of the uncertainty in the 0.5 MeV energy region on those results and on the analysis of special low-energy calibration data taken at the end of the Gerda data taking. This procedure has been documented in detail in [31], for which the energy scale and resolution uncertainties have been estimated in the same energy region. The FWHM with uncertainty at 514 keV for each analysis data set is reported in Table 1. The adopted mean calibration bias $\delta _i$ is 0 keV, with a Gaussian uncertainty of 0.1 keV for all the 5 analysis data sets.

## Results

Figure 3 shows the fit model at the profile likelihood maximum superimposed to data from the 5 data sets. Additionally, the contributions from the signal (^85^Kr peak at 514 keV) and the background (linear background plus 511 keV peak) are separately shown with dashed lines. The fit yields a *p*-value of 0.33. The difference between data and best-fit model in units of standard deviation is shown below each data set.

The best-fit value and 68% C.L. interval of the parameter representing the ^85^Kr activity in LAr at the start of the Gerda Phase II data taking $t_0 = \text {25 December 2015}$ is:

$$\begin{aligned} A_0 = (0.36 \pm 0.03)\,\text {mBq/kg}, \end{aligned}$$

where the confidence interval boundaries have been estimated assuming Gaussianity of the likelihood around its maximum and include all systematic uncertainties discussed in Sect. 6. An exponential extrapolation at cryostat filling time $t = \text {17 December 2009}$ yields an activity of $(0.53 \pm 0.05)\,\text {mBq/kg}$.

To determine the impact of the systematic uncertainties, the analysis has been repeated by removing the Gaussian pull terms from the likelihood and fixing the value of the conversion factor $\xi$ and the energy scale and resolution nuisance parameters to their central values: the resulting activity is $(0.53 \pm 0.05)$ mBq/kg at cryostat filling time. It can be therefore deduced that the statistical contribution dominates the global uncertainty budget.

## Conclusions

The Gerda collaboration has measured the activity of ${\upbeta }$-emitting ^85^Kr isotopes in the atmospheric LAr batch deployed in the experiment, at cryostat filling time. This result has been achieved by constraining the rate of ${\upgamma }$-rays following the decay of excited ^85^Rb daughters, as seen by the HPGe detector array immersed in the liquid. This technique is made possible by the excellent ${\upgamma }$-spectroscopy capabilities of Gerda.

We find a significantly lower activity than the central value reported by the WARP collaboration: $(0.12 \pm 0.09)$ Bq/kg in atmospheric LAr [5]. Within experimental uncertainties, however, the two results are compatible. Our measured value is also lower than the one reported by the DarkSide collaboration in underground LAr: $(2.05 \pm 0.13)$ mBq/kg [6]. The latter, unexpectedly high, has been attributed to in situ contamination. Given the strong influence of the details of the processes of distillation and handling of gas and liquids obtained from the atmosphere, the ^85^Kr activity is typically subject to a high variability across different experiments.

The Large Enriched Germanium Experiment for Neutrinoless-${\upbeta }$ ${\upbeta }$ Decay collaboration is currently operating the LEGEND-200 experiment at LNGS, within the existing Gerda cryostat. About 200 kg of HPGe detectors, immersed in a fresh batch of atmospheric LAr, will offer the possibility to repeat the measurement.

## Data Availability

My manuscript has no associated data. [Authors’ comment: All relevant results are collected in Fig. 3. For further information contact the GERDA Collaboration (gerda-eb@mp-ihd.mpg.de)].

## Footnotes

1 In the following we use ‘activity’ in the sense of ‘specific activity’.

2 https://www.linde-gas.it.

## Code Availability

My manuscript has no associated code/software. [Author’s comment: No code/software was generated or analysed during the current study.]
